# Depressive Symptoms Increase the Risk of Mortality for White but Not Black Older Adults

**DOI:** 10.3390/healthcare6020036

**Published:** 2018-04-23

**Authors:** Shervin Assari

**Affiliations:** 1 Department of Psychiatry, University of Michigan, Ann Arbor, MI 48109, USA; assari@umich.edu; Tel.: +734-232-0445; Fax: +734-615-8739; 2 Center for Research on Ethnicity, Culture and Health, School of Public Health, University of Michigan, Ann Arbor, MI 48109, USA

**Keywords:** race, ethnic groups, African Americans, mortality, depression, depressive symptoms

## Abstract

*Introduction.* Long-term studies have shown that depressive symptoms predict the risk of mortality. However, it is unknown if this effect is present in shorter time intervals. In addition, recent research suggests that the salience of the negative affect on the risk of mortality is not similar across racial groups. The current study uses data from a national study of Black and White older adults to examine racial differences in the effect of baseline depressive symptoms on mortality risk over three years in the United States. *Methods.* This study used a longitudinal prospective design and followed 1493 older adults who were either White (*n* = 759) or Black (*n* = 734) for three years from 2001 to 2004. Depressive symptoms measured at baseline was the independent variable. Demographic factors, socio-economic characteristics (education, income, marital status), health behaviors (smoking and drinking), and health (self-rated health) measured at baseline in 2001 were covariates. The dependent variable was all-cause mortality between 2001 and 2004. Race was the moderator. Logistic regressions were used for data analysis. *Results.* In the pooled sample, high depressive symptoms at baseline were not associated with the three-year risk of mortality. In the pooled sample, we found a significant interaction between race and depressive symptoms on mortality, suggesting a stronger effect for Whites in comparison to Blacks. In race stratified models, depressive symptoms at baseline were predictive of mortality risk for Whites, but not Blacks. *Conclusions*. In the United States, Black-White differences exist in the effects of depressive symptoms on mortality risk in older adults. White older adults may be more vulnerable to the effects of depressive symptoms on mortality risk.

## 1. Introduction

Psychosocial resources (e.g., education, income, employment, and marital status) and psychological assets (e.g., affect and coping) are essential for maintaining health and well-being [1,2]. Individuals with high levels of psychosocial risk factors are at a higher risk of poor health [3,4], physical functioning [5], chronic disease [6], and mortality [7]. Negative affect and depressive symptoms also increase the risk of mortality [8,9].

In a number of studies, race, ethnicity, and class have been found to alter the effects of psychosocial resources and assets on chronic disease [10] and health [11]. Race, ethnicity, and class also mitigate the effects of depression on physical health [12,13,14,15,16,17,18,19]. Racial and ethnic groups differ in separate [20] and combined [21] effects of depression and anxiety on obesity, [20,21,22] cardiovascular diseases (CVDs) [11,22,23,24], and well-being [12]. All this literature suggests that race and ethnicity operate as moderators for the effects of psychosocial factors on physical health outcomes [15,16,17,18,19,24,25,26,27].

Although Cooper et al. [28], Lewis et al. [11,23], Assari et al. [22,24,29], and Capistrant et al. [30] have all documented Black-White differences in the effect of depression on coronary artery disease (CAD) and CAD risk factors, the results of these studies are inconsistent. Cooper et al. showed that among individuals with depression, comorbid Posttraumatic Stress Disorder (PTSD) was linked to a lower and higher risk of CAD for Whites and Blacks, respectively [28]. In a longitudinal study by Lewis et al., high depressive symptoms predicted CVD and stroke mortality for Blacks but not Whites [11]. In another prospective study by Capistrant et al., race did not modify the effect of depression on CVD mortality [30].

Racial differences are not specific to the effects of depressive symptoms [8,9,31,32,33] as similar patterns are shown for a wide range of other psychosocial factors such as mastery [33,34], sleep [35], and perceived health [36]. The effects of education [37], income [38], employment [39], neighborhood quality [40], and social network [41] are shown to be stronger for Whites than Blacks. It is, however, not only race, but also SES, that buffers the effects of these resources and assets on mortality [7]. As race and social class have a strong overlap [42], it is still unknown whether it is race or SES that moderates the effects of these risk and protective factors.

To replicate and extend the results of the previous studies, we conducted this study to compared Black and White older adults in the United States (USA) for the effects of depressive symptoms on the short-term risk of mortality over a three year period.

## 2. Methods

### 2.1. Design and Setting

Religion, Aging, and Health Survey, 2001–2004, was a longitudinal panel study of older adults in the United States with three years of follow-up. We used data from Wave 1 and Wave 2 of the panel study [43].

### 2.2. Ethics

The Religion, Aging, and Health Survey protocol was approved by the University of Michigan Institutional Review Board (IRB). All participants provided informed consent.

### 2.3. Participants

The current study only included Black or White older adults. The sample was limited to non-institutionalized, English-speaking individuals, 65 years old or older at the time of enrollment. The study sampling was restricted to the coterminous (not Contiguous) United States (i.e., not including Alaska and Hawaii). The study sample was limited to Christians or those who were never associated with any faith. The study oversampled Blacks, so almost half of the sample is Black [43].

### 2.4. Sampling Frame

The study used random sampling to recruit a national sample. The sampling frame consisted of all eligible individuals in the Medicare Beneficiary list that were maintained by the Centers for Medicare and Medicaid Services (CMS) at the time of survey in 2001 [44]. The study used a five-step sampling process to draw individuals from the CMS file.

### 2.5. Data Collection

Data were collected by Louis Harris and Associates (now Harris Interactive, New York, NY, USA). Wave 1 interviews were performed between March and August of 2001 [43].

### 2.6. Measures

Race, demographic factors (age and gender), SES (education, income, and marital status), depressive symptoms, health risk behaviors (smoking and drinking), and health (self-rated health) were measured at baseline in 2001.

*Sociodemographic Factors.* Demographic factors were age (continuous measure) and gender (1 female 0 male). Socioeconomic characteristics included educational level (high school diploma 1 lower education 0), marital status (married 1 versus others 0), and income of the respondent (10 level categorical variable). Higher scores were indicative of higher SES.

*Health Behaviors.* Data were collected on self-reported history of smoking and drinking. We used dichotomous variables for smoking (current smoker = 1, never or ex-smoker = 0) and drinking (1 = current drinker and 0 = non-drinker). Single-item measures were previously used to measure smoking and drinking [45].

*Self-rated health (poor).* Individuals were asked a single question: “How would you rate your overall health at the present time?” Response items included: (1) Excellent, (2) Good, (3) Fair, and (4) Poor. We dichotomized the responses to excellent to fair (0) or poor (1). This single-item measure has shown high reliability and validity for the prediction of all-cause mortality of adults.

*Depressive Symptoms.* An eight-item version of the Center for Epidemiological Studies-Depression scale (CES-D) [46] was used to measure depressive symptoms. Respondents were asked about their negative emotions such as (1) blues, (2) felt depressed, (3) crying spells, (4) feeling sad, (5) not feel like eating (poor appetite), (6) feeling that everything is an effort, (7) restless sleep, and (8) could not get going. These items measure the negative affect and somatic symptoms. The eight-item CES-D measure has shown acceptable reliability and validity [47] as compared to the original 20-item CES-D measure [48,49,50]. Response items ranged from “rarely or none” (score 1) to “most or all of the time” (score 4). A mean score was calculated with a potential range from 1 to 4. This measure was operationalized as a continuous measure, with a higher score indicating more depressive symptoms (Cronbach Alpha = 0.87 for all, 0.85 for Whites, 0.89 for Blacks).

*Mortality.* Mortality data were obtained through various sources including the national death index, the death certificate, and the informants. Mortality was operationalized as a dichotomous variable (1 deceased, 0 alive). Mortality during the three year follow-up period was considered regardless of its time and cause. From all 1493 participants, 208 individuals were deceased during the follow up period.

### 2.7. Statistical Analysis

We used SPSS 22.0 for data analysis. Mean (SD) and frequency tables were used to describe the sample overall, and by race. We used logistic regressions in the pooled sample, and specific to race, for our multivariable models. In all models, depressive symptoms were the independent variable. All-cause mortality over the three year follow up period was the dependent variable. Demographic factors, socio-economic characteristics, health risk behaviors, and health at baseline were the covariates. Race was the focal moderator. We reported odds ratios (OR), associated 95% confidence intervals (CIs), and *p* values. *p* values less than 0.05 were considered as statistically significant.

## 3. Results

### 3.1. Sample

The study followed 1493 individuals for three years. All participants were older adults (age 65 or older). This sample was either Black (*n* = 734) or White (*n* = 759). From the 1493 participants, 208 individuals were deceased during follow up. From all the individuals who were deceased over the follow up period, 112 (54%) were Black and 96 (46%) were White.

### 3.2. Descriptive Statistics

Table 1 summarizes the descriptive statistics at baseline for the overall sample, as well as based on race. While age was similar between Black and White individuals, Blacks and Whites differed in gender, as the Black sample had a higher composition of females than Whites. Black participants also had a lower education, lower income, and were less frequently married. Blacks were smokers more frequently than Whites. However, they were less likely to be a drinkers, compared to Whites. Compared to Whites, Blacks had poorer self-rated health. Depressive symptoms were also higher among Black than White participants.

### 3.3. Models in the Pooled Sample

Table 2 shows the results of two logistic regressions in the pooled sample. While depressive symptoms were not a predictor of mortality in the pooled sample (*Model 1*), we found a significant interaction between race and depressive symptoms on mortality, suggesting a larger effect of depressive symptoms on mortality for Whites, compared to Blacks (*Model 2*).

### 3.4. Models by Race

Table 3 summarizes the results of two race-specific models to estimate the association between depressive symptoms and the subsequent risk of mortality among Blacks and Whites. *Model 3* showed that, in Whites, depressive symptoms at baseline were associated with an increased risk of mortality. *Model 4*, however, showed that this association could not be found for Blacks.

## 4. Discussion

The current study had two findings. Based on the first finding, baseline depressive symptoms did not predict the three-year mortality risk for older adults in the overall sample. Based on our second finding, however, race altered this association. High depressive symptoms at baseline increased the short-term risk of mortality for White but not Black older adults. The second finding replicates some recent findings on other cohorts, age groups, and follow up durations [8,9,51].

In a 25-year follow up study of Black and White adults, [8] high depressive symptoms at baseline were predictive of mortality risk for Whites but not Blacks [8]. The same pattern is shown for the effects of depressive symptoms and anger on mortality due to heart disease [9] and renal disease [51]. This is not because our tools do not correctly measure depressive symptoms in Blacks [52,53]. It is possible because Blacks who are depressed maintain high levels of positive emotions that can potentially undo the harmful physiological effects of negative affect [54,55].

Depressive symptoms [56] and neuroticism [57] predict long-term risk of MDD for Whites, but not Blacks. Black-White differences in the predictive role of negative affect on future risk of MDD suggests that a single measurement of negative affect is not sufficient to evaluate the future risk of MDD for Black individuals. Depressive symptoms and neuroticism, however, reflect the future risk of depression for Whites very well [56,57].

Racial and ethnic differential effects are not limited to the effects of depression. In line with findings on negative affect [8,9,31,32,33], health effects of mastery [33,34], sleep [35], and perceived health [36] are all larger for Whites, compared to Blacks. The same pattern also holds for the health effects of education [37], income [38], employment [39], neighborhood quality [40], and social network [41], which are all larger for Whites than Blacks. These findings collectively suggest that the health effects of psychosocial factors are not universal across all racial groups, and may be race-specific.

The above differential effects align with the Minorities’ Diminished Return Theory [58,59], defined as smaller health effects of economic resources and psychological assets for the minority compared to the majority population. Given that existing racism adds societal barriers to the lives of minority individuals, the very same resources and assets (and lack thereof) show smaller effects on the lives of Blacks than Whites. Blacks experience a decline in health regardless of their baseline mental health [60], Blacks develop MDD regardless of their previous depressive symptoms [56,57], and Blacks develop poor health outcomes regardless of their economic status [38,61,62,63]. These should be seen as a systemic inequality in health gain from resources and assets [58,59]. Unless these differential gains are addressed, the existing racial gap between the health of Blacks and Whites may widen.

### Limitations

Our study had a few limitations. First, the database was old, dating from 2001–2004. However, the results are in line with other studies that have used more recent data [8,9,51]. Demographic and socio-political changes are extremely important, and impact findings related to race and minority status. Second, the validity of depressive symptoms and negative affect may depend upon race. In addition, the current study failed to control for some important confounders such as baseline medical conditions, stress, and access to and use of health care. Finally, the study only included Blacks and Whites. Future research should include other racial groups. A major strength of this study was the enrollment of a national sample.

## 5. Conclusions

In summary, depressive symptoms increase the risk of mortality over a three-year period in White but not Black older adults. This finding is in line with other psychosocial constructs showing health effects that are not universal but specific to race. This finding also has implications for healthcare and public health.

## Figures and Tables

**Table 1:** Descriptive statistics in the pooled sample and by race.

Characteristics	All (*n* = 1439)		Whites (*n* = 1439)		Blacks (*n* = 734)	
	Mean	SD	Mean	SD	Mean	SD
Age	75.14	6.67	75.37	6.82	74.91	6.49
Income *	4.59	2.49	5.63	2.49	3.49	1.96
Depressive Symptoms *	1.56	0.62	1.54	0.59	1.59	.65
	n	%	n	%	n	%
Gender *						
Male	573	38.20	314	41.37	256	34.88
Female	927	61.80	445	58.63	478	65.12
Education *						
Low	609	40.98	200	26.60	407	55.98
High	877	59.02	552	73.40	320	44.02
Married *						
No	778	52.28	306	40.53	467	64.33
Yes	710	47.72	449	59.47	259	35.67
Smoking *						
No	1342	89.59	698	92.08	638	87.04
Yes	156	10.41	60	7.92	95	12.96
Drinking *						
No	1030	68.76	451	59.50	574	78.31
Yes	468	31.24	307	40.50	159	21.69
SRH Poor *						
No	1322	88.37	694	91.80	622	84.86
Yes	174	11.63	62	8.20	111	15.14

Source: Religion, Aging, and Health Survey, 2001–2004. * *p* < 0.05.

**Table 2:** Association between baseline depressive symptoms (2001) and all-cause mortality (2001–2004) using logistic regression in the pooled sample (*n* = 1439).

Characteristics	All (*n* = 1439) *Model 2*		All (*n* = 1439) *Model 1*	
	OR	95% CI	OR	95% CI
Race (Black)	0.96	0.63–1.47	2.37	0.83–6.73
Age	1.07 ***	1.05–1.10	1.07 ***	1.05–1.10
Gender (Female)	0.61 *	0.40–0.94	0.62 *	0.40–0.96
Education	1.46 #	0.94–2.27	1.47 #	0.95–2.28
Marital Status (Married)	0.83	0.52–1.31	0.84	0.53–1.32
Income	0.92	0.83–1.03	0.93	0.83–1.03
Smoking	0.80	0.43–1.49	0.83	0.45–1.56
Drinking	0.81	0.51–1.29	0.84	0.53–1.34
Self-Rated Health (SRH)	3.96 ***	2.40–6.52	4.12 ***	2.50–6.80
Depressive Symptoms	1.18	0.87–1.58	1.58 *	1.03–2.42
Depressive Symptoms × race (Black)	-	-	0.59 *	0.34–1.00
Intercept	0.00 ***		0.00 ***	

Source: Religion, Aging, and Health Survey, 2001–2004. # *p* < 0.1, * *p* < 0.05, ** *p* < 0.01, *** *p* < 0.001.

**Table 3:** Association between baseline depressive symptoms (2001) and all-cause mortality (2001–2004) using logistic regression among Whites (*n* = 759) and Blacks (*n* = 734).

Characteristics	Whites (*n* = 759) *Model 3*		Blacks (*n* = 734) *Model 4*	
	OR	95% CI	OR	95% CI
Age	1.08 **	1.04–1.12	1.08 **	1.03–1.12
Gender (Female)	0.70	0.38–1.30	0.56 #	0.30–1.03
Education	1.11	0.58–2.10	1.89 *	1.04–3.41
Marital Status (Married)	0.91	0.48–1.72	0.79	0.41–1.52
Income	0.93	0.81–1.07	0.95	0.80–1.12
Smoking	1.60	0.67–3.86	0.49	0.19–1.25
Drinking	0.95	0.51–1.77	0.73	0.35–1.51
Self-Rated Health (SRH)	2.54 *	1.17–5.53	5.98 **	3.05–11.72
Depressive Symptoms	1.74 *	1.12–2.68	0.87	0.57–1.34
Intercept	0.00 **		0.00 **	

Source: Religion, Aging, and Health Survey, 2001–2004. # *p* < 0.1, * *p* < 0.05, ** *p* < 0.001.

## References

[1] Ross C., Sastry J., Aneshensel C.S., Phelan J.C. (1999). The sense of personal control: Social-structural causes and emotional consequences. Handbook of the Sociology of Mental Health.

[2] Lachman M.E., Neupert S.D., Agrigoroaei S., Schaie K.W., Willis S.L. (2011). The relevance of control beliefs for health and aging. Handbook of the Psychology of Aging.

[3] Mirowsky J., Ross C.E., Horwitz A.V., Scheid T.L. (1999). Well-being across the life course. A Handbook for the Study of Mental Health: Social Contexts, Theories, and Systems.

[4] Keeton C.P., Perry-Jenkins M., Sayer A.G. (2008). Sense of control predicts depressive and anxious symptoms across the transition to parenthood. J. Fam. Psychol..

[5] Infurna F.J., Gerstorf D., Zarit S.H. (2011). Examining dynamic links between perceived control and health: Longitudinal evidence for differential effects in midlife and old age. Dev. Psychol..

[6] Surtees P.G., Wainwright W.J., Luben R., Wareham N.J., Bingham S., Khaw K.T. (2010). Mastery is associated with cardiovascular disease mortality in men and women at apparently low risk. Health Psychol..

[7] Turiano N.A., Chapman B.P., Agrigoroaei S., Infurna F.J., Lachman M. (2014). Perceived control reduces mortality risk at low, not high, education levels. Health Psychol..

[8] Assari S., Moazen-Zadeh E., Lankarani M.M., Micol-Foster V. (2016). Race, Depressive Symptoms, and All-Cause Mortality in the United States. Front. Public Health.

[9] Assari S. (2017). Hostility, Anger, and Cardiovascular Mortality among Blacks and Whites. Res. Cardiovasc. Med..

[10] Assari S. (2016). Race and Ethnic Differences in Additive and Multiplicative Effects of Depression and Anxiety on Cardiovascular Risk. Int. J. Prev. Med..

[11] Lewis T.T., Guo H., Lunos S., Mendes de Leon C.F., Skarupski K.A., Evans D.A., Everson-Rose S.A. (2011). Depressive symptoms and cardiovascular mortality in older black and white adults: Evidence for a differential association by race. Circ. Cardiovasc. Qual. Outcomes.

[12] Assari S. (2014). Separate and combined effects of anxiety, depression and problem drinking on subjective health among black, Hispanic and non-Hispanic white men. Int. J. Prev. Med..

[13] Assari S., Lankarani M.M., Lankarani R.M. (2013). Ethnicity modifies the additive effects of anxiety and drug use disorders on suicidal ideation among black adults in the United States. Int. J. Prev. Med..

[14] Assari S. (2014). Synergistic effects of lifetime psychiatric disorders on suicidal ideation among blacks in the USA. J. Racial Ethn. Health Disparities.

[15] Assari S. (2014). The link between mental health and obesity: Role of individual and contextual factors. Int. J. Prev. Med..

[16] Assari S. (2014). Chronic medical conditions and major depressive disorder: Differential role of positive religious coping among African Americans, Caribbean blacks and non-Hispanic whites. Int. J. Prev. Med..

[17] Assari S. (2013). Race and ethnicity, religion involvement, church-based social support and subjective health in United States: A case of moderated mediation. Int. J. Prev. Med..

[18] Krok-Schoen J.L., Baker T.A. (2014). Race differences in personality and affect between older white and black patients: An exploratory study. J. Racial Ethn. Health Disparities.

[19] Bruce M.A., Beech B.M., Hamilton G.E., Collins S.M. (2014). Knowledge and perceptions about clinical trial participation among African American and Caucasian College Students. J. Racial Ethn. Health Disparities.

[20] Assari S. (2014). Association between obesity and depression among American blacks: Role of ethnicity and gender. J. Racial Ethn. Health Disparities.

[21] Assari S. (2014). Additive effects of anxiety and depression on body mass index among blacks: Role of ethnicity and gender. Int. Cardiovasc. Res. J..

[22] Assari S., Caldwell C.H. (2015). Gender and ethnic differences in the association between obesity and depression among black adolescents. J. Racial Ethn. Health Disparities.

[23] Lewis T.T., Everson-Rose S.A., Colvin A., Matthews K., Bromberger J.T., Sutton-Tyrrell K. (2009). Interactive effects of race and depressive symptoms on calcification in African American and white women. Psychosom. Med..

[24] Assari S. (2014). Race and ethnic differences in associations between cardiovascular diseases, anxiety, and depression in the United States. Int. J. Travel Med. Glob. Health.

[25] Wysocki J., Newby C., Balart L., Shores N. (2014). HCV triple therapy is equally effective in African-Americans and Non-African-Americans. J. Racial Ethn. Health Disparities.

[26] Insaf T.Z., Shaw B.A., Yucel R.M., Chasan-Taber L., Strogatz D.S. (2014). Lifecourse Socioeconomic Position and Racial Disparities in BMI Trajectories among Black and White Women: Exploring Cohort Effects in the Americans Changing Lives’ Study. J. Racial Ethn. Health Disparities.

[27] Hicken M.T., Lee H., Mezuk B., Kershaw K.N., Rafferty J., Jackson J.S. (2013). Racial and ethnic differences in the association between obesity and depression in women. J. Women’s Health.

[28] Cooper D.C., Trivedi R.B., Nelson K.M., Reiber G.E., Beaver K.A., Eugenio E.C., Fan V.S. (2014). Post-traumatic Stress Disorder, Race/Ethnicity, and Coronary Artery Disease among Older Patients with Depression. J. Racial Ethn. Health Disparities.

[29] Assari S., Sonnega A. (2017). Racial Differences in the Predictive Role of High. Depressive Symptoms on Incident Heart Disease over 18 Years: Results from the Health and Retirement Study. Res. Cardiovasc. Med..

[30] Capistrant B.D., Gilsanz P., Moon J.R., Kosheleva A., Patton K.K., Glymour M.M. (2013). Does the association between depressive symptoms and cardiovascular mortality risk vary by race? Evidence from the Health and Retirement Study. Ethn. Dis..

[31] Assari S., Lankarani M.M., Burgard S.A. (2016). Black White Difference in Long Term Predictive Power of Self-Rated Health on All-Cause Mortality in United States. Ann. Epidemiol..

[32] Assari S., Burgard S.A., Zivin K. (2015). Long Term Reciprocal Associations between Depression and Chronic Medical Conditions; Longitudinal Support for Black-White Health Paradox. J. Racial Ethn. Health Disparities.

[33] Assari S., Lankarani M.M. (2016). Chronic Medical Conditions and Negative Affect; Racial Variation in Reciprocal Associations over Time. Front. Psychiatry.

[34] Assari S. (2017). Race, sense of control over life, and short-term risk of mortality among older adults in the United States. Arch. Med. Sci..

[35] Assari S., Sonnega A., Leggett A., Pepin R.L. (2017). Residual Effects of Restless Sleep over Depressive Symptoms on Chronic Medical Conditions: Race by Gender Differences. J. Racial Ethn. Health Disparities.

[36] Assari S. (2017). General Self-Efficacy and Mortality in the USA; Racial Differences. J. Racial Ethn. Health Disparities.

[37] Assari S., Lankarani M.M. (2016). Race and Urbanity Alter the Protective Effect of Education but not Income on Mortality. Front. Public Health.

[38] Assari S. (2018). The Benefits of Higher Income in Protecting against Chronic Medical Conditions Are Smaller for African Americans than Whites. Healthcare.

[39] Assari S., Assari S. (2018). Life Expectancy Gain Due to Employment Status Depends on Race, Gender, Education, and Their Intersections. J. Racial Ethn. Health Disparities.

[40] Assari S. (2016). Perceived Neighborhood Safety Better Predicts 25-year Mortality Risk among Whites than Blacks. J. Racial Ethn. Health Disparities.

[41] Assari S. (2017). Whites but Not Blacks Gain Life Expectancy from Social Contacts. Behav. Sci..

[42] Drake K.A., Galanter J.M., Burchard E.G. (2008). Race, ethnicity and social class and the complex etiologies of asthma. Pharmacogenomics.

[43] Krause N. (2006). Religion, Aging, and Health Survey, 2001, 2004 [United States] ICPSR03255-v2.

[44] Centers for Medicare & Medicaid Services Medicare Beneficiary Characteristics. https://www.cms.gov/Research-Statistics-Data-and-Systems/Statistics-Trends-and-Reports/Chronic-Conditions/Medicare_Beneficiary_Characteristics.html.

[45] Harvey I.S., Alexander K. (2012). Perceived social support and preventive health behavioral outcomes among older women. J. Cross Cult. Gerontol..

[46] Radloff L.S. (1977). The CES-D scale: A self-report depression scale for research in the general population. Appl. Psychol. Meas..

[47] Van de Velde S., Levecque K., Bracke P. (2009). Measurement equivalence of the CES-D 8 in the general population in Belgium: A gender perspective. Arch. Public Health.

[48] Amtmann D., Kim J., Chung H., Bamer A.M., Askew R.L., Wu S., Cook K.F., Johnson K.L. (2014). Comparing CESD-10, PHQ-9, and PROMIS depression instruments in individuals with multiple sclerosis. Rehabil. Psychol..

[49] Andresen E.M., Malmgren J.A., Carter W.B., Patrick D.L. (1994). Screening for depression in well older adults: Evaluation of a short form of the CES-D (center for epidemiologic studies depression scale). Am. J. Prev. Med..

[50] Zhang W., O’Brien N., Forrest J.I., Salters K.A., Patterson T.L., Montaner J.S., Hogg R.S., Lima V.D. (2012). Validating a shortened depression scale (10 item CES-D) among HIV-positive people in British Columbia, Canada. PLoS ONE.

[51] Assari S., Burgard S.A. (2015). Black-White differences in the effect of baseline depressive symptoms on deaths due to renal diseases: 25 year follow up of a nationally representative community sample. J. Renal Inj. Prev..

[52] Assari S., Moazen-Zadeh E. (2016). Confirmatory Factor Analysis of the 12-Item Center for Epidemiologic Studies Depression Scale among Blacks and Whites. Front. Psychiatry.

[53] Assari S., Moazen-Zadeh E. (2016). Ethnic Variation in the Cross-sectional Association between Domains of Depressive Symptoms and Clinical Depression. Front. Psychiatry.

[54] Lankarani M.M., Assari S. (2017). Positive and Negative Affect More Concurrent among Blacks than Whites. Behav. Sci..

[55] Assari S., Lankarani M.M. (2016). Depressive Symptoms Are Associated with More Hopelessness among White than Black Older Adults. Front. Public Health.

[56] Moazen-Zadeh E., Assari S. (2016). Depressive Symptoms Predict Major Depressive Disorder after 15 Years among Whites but Not Blacks. Front. Public Health.

[57] Assari S. (2017). Neuroticism Predicts Subsequent Risk of Major Depression for Whites but Not Blacks. Behav. Sci..

[58] Assari S. (2017). Unequal Gain of Equal Resources across Racial Groups. Int. J. Health Policy Manag..

[59] Assari S. (2018). Health disparities due to diminished return among black Americans: Public policy solutions. Soc. Issues Policy Rev..

[60] Assari S., Burgard S., Zivin K. (2015). Long-Term Reciprocal Associations between Depressive Symptoms and Number of Chronic Medical Conditions: Longitudinal Support for Black-White Health Paradox. J. Racial Ethn. Health Disparities.

[61] Assari S. (2017). Combined Racial and Gender Differences in the Long-Term Predictive Role of Education on Depressive Symptoms and Chronic Medical Conditions. J. Racial Ethn. Health Disparities.

[62] Assari S. (2017). Social Determinants of Depression: The Intersections of Race, Gender, and Socioeconomic Status. Brain Sci..

[63] Assari S., Nikahd A., Malekahmadi M.R., Lankarani M.M., Zamanian H. (2017). Race by Gender Group Differences in the Protective Effects of Socioeconomic Factors against Sustained Health Problems across Five Domains. J. Racial Ethn. Health Disparities.

